# Hand games, naughty games

**DOI:** 10.11604/pamj.2020.35.132.20704

**Published:** 2020-04-20

**Authors:** Kawtar Belkhadir, Ouafaa Cherkaoui

**Affiliations:** 1 Departement d'Ophtalmologie Unit A, Hôpital des Spécialités, Faculté de Médicine et Pharmacie, Université Mohammed V, Rabat, Maroc

**Keywords:** Eyelid wound, skin graft, post traumatic wound, reconstructive surgery

## Image in medicine

We report the case of a 13-year-old child who presented to the ophthalmic emergencies for left eye trauma because of a sore eyelid, while fighting with one of his classmates using pairs of scissors. The clinical examination found a wound on the upper left eyelid with loss of substance of the skin without involvement of the orbicularis muscle (A). Examination of the left eye found a visual acuity preserved at 12/10 without optical correction, a calm anterior segment and a flat retina. Faced with the loss of cutaneous substance, it was decided to perform a total skin graft from the abdominal skin (B). The sample was taken using a scalpel and the graft was defatted. The total skin graft was immediately performed, and sutured with fluted edges to prevent healing in the staircase (C). The patient was seen again 10 days after the graft, with good budding of the graft, and removal of the stitches (D). Fat bandage care has been recommended to allow good healing of the graft. Eyelid sores in children represent a multitude of clinical forms. These wounds require early and appropriate management, with a complete initial clinical examination, to look for lesions of the free edge or lachrymal passages, but also a careful examination of the globe in order to rule out a wound of the globe that would be associated. Their management should be early and specialized, in order to avoid the occurrence of skin retraction, or harmful aesthetic sequelae.

**Figure 1:**
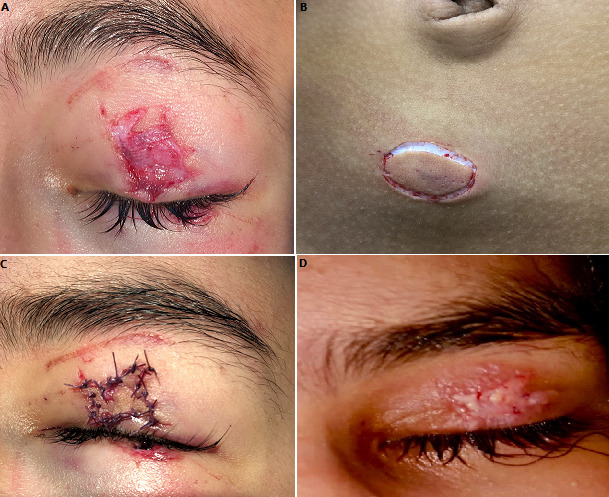
A) initial appearance of the eyelid sore; B) site of the graft used; C) aspect after suture of the graft edges to edges; D) appearance after removal of the stitches

